# Study on the comprehensive treatment of migraine with traditional Chinese medicine based on the new pathophysiological mechanism: A review

**DOI:** 10.1097/MD.0000000000039487

**Published:** 2024-08-30

**Authors:** Yue Shen, Zeguang Li, Jing Wang, Zitong Qiu

**Affiliations:** a Graduate School, Heilongjiang University of Chinese Medicine, Harbin, Heilongjiang Province, China; b First Affiliated Hospital, Heilongjiang University of Chinese, Harbin, Heilongjiang Province, China.

**Keywords:** CGRP, CSD, migraine, no aura, traditional Chinese medicine

## Abstract

Migraine is a ubiquitous neurological disorder that affects approximately 1 billion people worldwide. Migraine is the second leading cause of illness in people of all ages worldwide. Uncertainty in migraine diagnosis leads to unnecessary testing and increases the treatment costs. To date, the pathogenesis of migraine is not fully understood, but it is generally believed that migraine involves the trigeminal nerve and its axonal projections to intracranial blood vessels. Pain signals from the trigeminal neurovascular system are transmitted to the brain, resulting in migraines. As an important component of complementary and alternative medicine, traditional Chinese medicine (TCM) has shown significant efficacy in the treatment of migraine, and has attracted increasing attention worldwide. This review is based on the pathophysiology of migraines in modern medicine. To explore the comprehensive treatment of migraine using TCM, acupuncture, and various other TCM treatments.

## 1. Introduction

Migraine is a polygenic neurological disorder with a high incidence and is ranked the second leading cause of disability worldwide.^[1]^ The pathogenesis of migraine is highly heritable, and if one parent has migraine, the probability of children suffering from migraine is 75%.^[2,3]^ Migraines can come on at any time, but usually come in late childhood, early adolescence, or mid-adulthood. Migraine was reported as early as 1935, when it was called “hypoglycemic pain.”^[4]^ In a review published by Willem Amery in 1982, it was proposed that metabolism is related to migraine pathogenesis.^[5]^ Migraine is a chronic and often lifelong condition that directly affects more than 1 billion people worldwide.^[6]^ Migraine poses a significant health burden to healthcare systems worldwide because of the relatively limited availability and high cost of modern medical treatment. Chinese medicine, as a complementary and alternative medicine, has rich experience and remarkable clinical efficacy in the treatment of migraine. Therefore, based on the pathophysiological mechanism of migraine, this study explored the treatment of migraine with traditional Chinese medicine (TCM) combined with acupuncture.

## 2. Retrieval strategy and selection criteria

We searched the PubMed and CNKI databases (from the establishment of the database to August 2023) for original studies, literature reviews, systematic reviews, and meta-analyses. The search keywords were “migraine,” “pathophysiology,” “traditional Chinese medicine,” “acupuncture and moxibustion,” “combination of acupuncture and medicine,” and the above search terms were used in a certain order. We mainly chose publications from the past 5 years, but we do not exclude earlier authoritative publications. At the same time, we also reviewed the bibliography of the articles that met the inclusion criteria and screened them to select relevant articles. There were no language restrictions on the search policies.

## 3. Epidemiology

To date, epidemiological studies on migraine have focused on the incidence rate, which is 18.2 cases per thousand in women, according to the American Migraine Prevalence and Prevention Study,^[7]^ and the peak age of onset is 20 to 24 years. The incidence rate is 6.2 cases per 1000 people, and the peak age of onset is 15 to 19 years old. According to a longitudinal study in Denmark,^[8]^ the overall incidence of migraine was 8.1 cases per 1000 people without migraine initially, 23 cases per 1000 women and 10 cases per 1000 men.

The 1-year prevalence of migraine has been frequently reported.^[9]^ The global one-year incidence rate is 15%, with the highest in Southeast Asia, 25% to 35%,^[10]^ and the lowest in China, 9%.^[11]^ According to a systematic review of the Global Burden of Disease Study, the global age-standardized incidence rate was 14.4%.^[11]^

The incidence of migraine varies from region to region, with the highest incidence in Nepal and the lowest in China, according to the Lancet Global Burden of Disease study.^[11]^ There are also sex differences in the incidence of migraines. According to a study by Chaojie Wang^[12]^ and others, there are significant differences in the changes in brain structure and function between men and women. In addition, a study using high-field magnetic resonance imaging found that women with migraine had specific changes in brain structure in the insular and precuneus regions, whereas men had specific changes in the parahippocampal gyrus.^[13–15]^ In addition, male migraine patients are more likely to be sensitive to painful stimuli than female migraine patients or healthy subjects, whereas females respond negatively to stimuli.^[16]^

## 4. Classification and characteristics

Medical history is the main basis for the diagnosis of migraine,^[17]^ and the clinical features of typical migraine include unilateral recurrent headache, pulsatile headache, moderate or severe headache, and symptoms such as nausea, vomiting, photophobia, and phonophobia. Although migraine pain is usually considered to be one-sided, approximately 40% of patients report bilateral headaches.^[18]^ Thus, the International Classification of Headache Disorders, third edition has defined clinical criteria for migraine without aura, migraine with aura, and rarer subtypes of migraine. Aura occurs in about one-third of migraine patients, and migraine with aura is characterized by transient focal neurological symptoms that gradually recur over a period of 5 to 60 minutes.^[17]^ The most common aura is visual, including changes in peripheral vision, such as flickering light, enlargement of the blind spot, flickering edges, or jagged lines. Common nonvisual auras include unilateral, diffuse numbness or tingling affecting the face and arms and thinking or language disorders. Usually, the headache symptoms of migraine begin within 1 hour after the aura, but some aura attacks do not develop into headaches.^[17]^ Another important aspect of migraine classification is CM diagnosis of chronic migraine. The progression of migraine to chronic migraine is associated with several potentially modifiable factors, including baseline headache frequency, overuse of acute medications, excessive caffeine intake, paresthesia, persistent and frequent nausea, obesity, sleep disturbances, and psychiatric disorders.^[19]^ International Classification of Headache Disorders, third edition defines chronic migraine as 15 or more headache days per month, at least 8 of which meet the clinical criteria for migraine with or without aura.^[17]^

The prodrome is more common than the aura. Prodrome is the first stage of migraine attack, which can begin hours or days before aura and headaches. Common prodromal symptoms include mood changes, irritability, fatigue, increased appetite, increased urge to urinate, difficulty concentrating, neck pain, and difficulty in falling asleep.

## 5. Genetic research

Phenotypic characteristics of migraine patients have been reported in a genomic study of migraine.^[20]^ Several genome-wide association studies (GWAS) have been conducted on migraine.^[20–22]^ In a GWAS study, a large sample of millions of common DNA variants, known as single nucleotide polymorphisms (SNPs), scattered throughout the genome was tested to determine their association with a trait. By determining the differences in allele frequencies between patients and controls, and following rigorous multiple testing, regions of the genome associated with disease can be identified. Global genomic studies have focused on headaches without aura,^[23]^ headache with aura,^[24]^ clinical samples,^[24]^ and population samples.^[22]^ Genes associated with these SNPs may be involved in neuronal, vascular, metalloproteinase, pain, and metal-iron related pathways.^[25]^ These GWAS also used pathway analysis methods, which showed a typical enrichment of genes in vascular pathways, highlighting the possible vascular etiology of migraine.^[20]^ Gupta et al showed that the etiology of migraine is related to SNP.^[26]^

Whether migraine with aura and migraine without aura are different diseases or whether the 2 subtypes are genetically related remains controversial. Previous observations suggest a continuum of severity in migraine subtypes, with migraine with aura being more severe but not etiologically distinct from migraine without aura.^[27]^ Clinical and epidemiological studies have shown that the main form of attack in patients with migraine changes over time.^[28]^ Family studies have shown that migraine subtypes co-occur in families.^[28,29]^ Initially, GWAS focused on migraine with and without aura, and the 2 migraine subtypes were found to have different genomic loci.^[24,30]^ These studies had relatively small sample sizes and focused only on SNP. In a polygenic risk score study, cases of migraine with aura and hemiplegic migraine had a higher genetic burden than migraine without aura.^[31]^ This may partially explain the lower incidence of migraine with aura and hemiplegic migraines. Therefore, it is conceivable that the onset of these subtypes requires a high genetic load. This study showed that the higher the polygenic risk score, the stronger the family history of migraine, and the younger the age of onset, the more subtypes of migraine with aura.^[31]^ This is consistent with a clinical study in the Netherlands, in which the stronger the family history of migraine, the younger the age of onset, the higher the frequency of onset, the longer the duration of medication, and the more pronounced the subtype of migraine with aura.^[32]^

A recent study by Biobank found that 28 genomic loci were associated with the generalized “headache” phenotype.^[33]^ It is important to note that among the newly identified loci, only 14 were previously identified in migraine genomic studies.^[20]^ This may be related to the fact that epidemiology has shown that migraine and tension headache coexist.^[34]^

## 6. Pathophysiology

Peripheral neurogenic inflammation was originally thought to trigger migraine. Although this role has been reexamined largely due to the failure of plasma protein extravasation blockers to treat migraine in randomized controlled trial (RCT),^[35,36]^ neurogenic inflammation associated with trigeminal nerve excitation is still under discussion.^[37]^ One hypothesis is that peripheral trigeminal ganglion neurons are sensitized, which subsequently sensitizes secondary neurons in the caudal trigeminal nucleus and upper cervical cord. It projects rostrally to the thalamic nuclei and key regions of the medulla oblongata, brainstem, and diencephalon.^[38]^ These studies provide reliable evidence for early brainstem involvement in nociceptive migraines. It is becoming increasingly clear that the initiation of migraine attacks is associated with intrinsic brain dysfunction in more central regions and possibly with external factors.^[39]^ Whether central dysfunction, which manifests very clearly in the aura phase, promotes central sensitization remains an interesting area of research.

Migraine is thought to be a periodic disorder of sensory thresholds, which theorizes that the hypothalamus plays an important role as a central facilitator of pain.^[40]^ This effect has been demonstrated by functional neuroimaging.^[41]^ During the aura phase of migraine, the hypothalamus-brainstem connections to the spinal trigeminal nucleus and dorsal pons are altered.^[42]^ Recent functional imaging studies have shown that altered connections between the hypothalamic and trigeminal spinal nuclei and cortical areas are associated with the development of chronic migraine.^[43,44]^

The role of the cerebral cortex in migraine was initially associated with the phenomenon of aura and its characteristic symptoms.^[45]^ The aura is thought to be caused by cortical spreading depression.^[46]^ This was indirectly confirmed by functional imaging study.^[47]^ Cortical spreading depressing can activate trigeminal neurovasculature through a calcitonin gene-related peptide (CGRP)-mediated mechanism.^[48,49]^ However, it is unlikely to cause migraine and may constitute an epiphenomenon of migraine.^[50]^ Many changes in the structure and function of cortical regions related to pain processing occur in patients during the ictal and interictal phases.^[51]^ During the ictal phase, the function of the cortical network changes, which reflects the cognitive, pain, and emotional symptoms of migraine.^[52]^ Studies in patients with migraine aura have shown differences in brain structure in migraine patients,^[53]^ functional connectivity (FC),^[53]^ cortical excitability,^[54,55]^ and pain modulation in visual pathways.^[56]^

However, evidence from neuroimaging studies is sometimes inconclusive^[57]^ and meta-regression analyses have failed to pinpoint specific changes in migraine.^[58]^ This suggests that further research is needed to elucidate the pathophysiology of migraine.

## 7. Cognition of TCM

Migraine belongs to the category of “headaches” in TCM. It is called “head wind” “in the Huangdi Internal Classic, and its etiology is related to the “wind” of TCM pathogenesis.^[59]^ “Clinical Guide to Medical Records” also points out that the liver is the unyielding viscus, and the liver is yin in form but Yang in function. This theory of analogy in TCM holds that the liver, as 1 of the 5 zang organs and 6 fu organs, dominates human emotions. Emotional abnormalities can cause liver failure, hyperactivity of liver yang, and adverse flow of qi and blood. This can eventually lead to migraines. According to Danxi Experiential Therapy head wind, “head wind belongs to the wind on the left,” which means that migraine often occurs on the left side of the head. This disease is caused by wind evils. The book proposes the use of TCMs, such as Schizonepeta, field mint, and other drugs for treatment. It is also recorded in the Complete Record of Sacred Benevolence that migraine is caused by pathogenic factors due to a deficiency of meridians and collaterals, and then causes pain on one side and special pain in the frontal horn.^[60]^ Li Gao, 1 of the 4 masters of the Jin and Yuan Dynasties, classified Taiyin headache and Shaoyin headache as migraine on the basis of predecessors. Danxi Experiential Therapy has also supplemented all kinds of menstruation-inducing drugs for headaches. These studies have provided a detailed examination of the etiology, pathogenesis, syndrome differentiation, and treatment of migraine, which has laid a solid foundation for the treatment of migraine in later generations.

## 8. Etiology and pathogenesis

The etiology of migraine is nothing more than internal cause, external cause, and neither internal nor external causes.^[61]^ TCM believes that the head is “the meeting of all Yang,” which means that the head is the place where the “Yang qi” Yang qi’ of the whole human body converges and dominates the growth and development of the whole human body.

If the qi movement of the head is kept unobstructed, the human body maintains its essence and spirit. When the meridian and collateral are blocked, it will Lear Yang fails to ascend, and turbid yin fails to descend. Therefore, head diseases followed. All 6 Yang pulses reached their heads from the meridians. The Shaoyang meridian is the most widely distributed meridian in the head, and the Jueyin and Shaoyang meridians are interiorly and exteriorly related to each other. Therefore, migraine attacks are closely related to the 6 Yang pulses, particularly in the Jueyin and Shaoyang meridians. According to the theory of latent pathogen, latent pathogens caused by various factors, such as phlegm retention, blood stasis, and retention of fire in the patient’s body, are in a relatively “stable” state with the body. When external adverse stimuli are applied to the body, or the body is weak, latent pathogens are triggered. Evil moves and blocks channels and collaterals. This affects the balance of qi, blood, yin, and yang in the viscera and causes migraine.^[59]^

## 9. TCM for migraine

For the treatment of migraine with TCM, refer to the expert consensus on diagnosis, prevention, and treatment of migraine, revised by the Chinese administration of traditional Chinese medicine. According to the scheme, the TCM syndrome types of migraine are divided into 5 types: ascendant hyperactivity of liver Yang, syndrome of phlegm turbidity invading the head, syndrome of static blood invading the head, syndrome of dual deficiency of qi and blood, and liver-kidney depletion.^[62]^ However, to date, no authoritative TCM guidance program has been developed. Therefore, in clinical treatment, the dialectical treatment of migraines is not the same.

Liu designed a RCT to observe the clinical efficacy of Ephedra and Aconite and Asarum Decoction in the treatment of migraine.^[63]^ Eighty patients with migraine were randomly divided into control and observation groups, with 40 patients in each group. The control group was treated with Toutong capsules. The observation group was treated with Ephedra Asarum decoction (Ephedra, aconite, and Asarum) on the basis of the control group. Clinical efficacy, headache score, headache attack frequency, duration of each attack, TCM syndrome score, Pittsburgh Sleep Quality Index score, and serum biochemical indicators (5-hydroxytryptamine [5-HT] and endothelin-1 [ET-1]) were compared between the 2 groups before and after treatment. Conclusion: Ephedra Aconite and Asarum Decoction can effectively relieve migraine symptoms, reduce the frequency and duration of attacks, and improve sleep quality.

Suqin et al designed an RCT. Guishao Gexiong decoction (cassia twig, white peony root, Pueraria root, chuanxiong root, Asarum, notoptetygium root, Corydalis rhizome, suberect spatholobus stem, wenyujin rhizome, tall gastrodis tuber, efficacy of cyathula root, and prepared licorice root) for migraine in the syndrome of congealing cold with blood stasis.^[64]^ Ninety patients with chronic migraine of cold coagulation and blood stasis type were randomly divided into the treatment and control groups. A total of 49 patients were included in the treatment group. There were 41 patients in the control group. Before and after treatment, the patients were assessed using the simplified McGill pain questionnaire, numeric rating scale, and emotion scale for pain intensity and emotion scores. Frequency, degree, duration of headache, and accompanying symptoms (head tingling, aggravation of cold, emotional anxiety, and mental burnout) were scored. In addition, the efficacy of TCM syndromes was compared between the 2 groups of patients with various indicators and comprehensive efficacy. Researchers found that the Guishao Gexiong Decoction can not only significantly reduce the number of migraine attacks with cold coagulation and blood stasis, but also shorten the duration of headache. The Guishao Gexiong Decoction can also improve fatigue, anxiety, depression, and other accompanying symptoms.

Zhang Li et al Designed RCT. Toufeng decoction (chrysanthemum flower, tall gastrodis tuber, gambir plant, shrub chastetree fruit, Ligusticum root, Ledebouriella root, Chuanxiong root, Cyperus, Corydalis rhizome, and libanotus) in migraine patients with syndrome of ascending hyperactivity of liver Yang, Serum hs-C-reactive protein (CRP) expression, and cerebral arterial blood flow velocity.^[65]^ Eighty patients with migraine who met the diagnostic criteria were randomly divided into the treatment and control groups. The treatment group was treated with Toufeng Decoction formula granules, 1 bag each time, 3 times a day, and water was taken after meals. The control group was administered 0. 2 g, twice a day, after meals, the course of treatment was 30 days. The total efficacy rate, TCM syndrome score, serum hs-CRP expression, and cerebral artery blood flow velocity were measured before and after treatment. Researchers found that Toufeng Decoction could significantly improve the clinical symptoms and TCM syndrome scores of migraine patients and reduce their serum hs-CRP levels.

Xu et al designed an RCT to analyze the effectiveness and adverse reactions of Zhengan Xifeng Tang in the treatment of migraine.^[66]^ Seventy-two patients with migraine who received treatment were selected for observation in this study. Patients were randomly divided into 2 groups. Patients in the control group were treated with conventional Western medicine (nimodipine and flunarizine hydrochloride tablets). Patients in the observation group were treated with Western medicine and Zhengan Xifeng Tang (Pueraria root, Chuanxiong root, chrysanthemum flower, paniculate swallowwort root, tall gastrodis tuber, notoptetygium root, schizonepeta, ledebouriella root, scorpion, angelica root, licorice root, Centipede, and Sanqi). The clinical efficacy, recurrence rate, and adverse reactions of the 2 groups were compared, and the duration of headache symptoms and number of headache attacks in the 2 groups were analyzed. Conclusion: The clinical efficacy of Zhengan Xifeng Tang in the treatment of migraine is significant, which can not only promote the improvement of disease-related symptoms and avoid the recurrence of the disease, but also will not lead to serious adverse reactions.

Yang Fan designed an RCT to observe the analgesic effect of the modified Banxia Baizhu Tianma Tang on acute migraine attacks.^[67]^ A total of 120 patients were randomly divided into observation and control groups, with 60 cases in each group. The patients in the control group were treated with flunarizine before sleep. The observation group was treated with modified Banxia Baizhu Tianma Tang (Poria, Corydalis rhizome, white Atractylodes rhizome, Pinellia rhizome in ginger juice, tall gastrodis tuber, chuanxiong root, bamboo shavings, and fresh ginger) orally. The analgesic effect and clinical efficacy of the 2 groups were compared, and differences in pain degree, pain duration, pain index, serum CRP, and interleukin-6 (IL-6) levels between the 2 groups were observed. Conclusion: Jiawei Banxia Baizhu Tianma Tang has a good analgesic effect on acute migraine attacks, reducing the number of pain attacks and shortening the duration of pain. Researchers believe that its mechanism of action may be related to the improvement in serum CRP and IL-6 levels.

Jiang et al designed an RCT to study the efficacy of Headache I in the treatment of migraine and the improvement of free radical disorders in patients.^[68]^ One hundred twenty patients with migraine were randomly divided into 2 groups. The control group was administered flunarizine hydrochloride capsules. The patients in the observation group were additionally treated with Chuanxiong root, Angelica root, Notoptetygium root, Asarum, Schizonepeta, Ledebouriella root, Sanqi, Tall gastrodis tuber, Typhonium rhizome, ground beetles, stiff silkorms, centipedes, scorpions, and licorice roots). The clinical effects in the 2 groups of patients after treatment were analyzed. Conclusion: Headache I can improve intracranial blood flow, resist free radical disorders, and do not increase adverse reactions.

Wang Zhaowei et al designed an RCT to observe the effect of Chuanxiong root, Angelica root, white peony root, Corydalis rhizome, common self-healing fruit-spike, shrub chastetree fruit, ligusticum root, field mint, chrysanthemum flower, bupleurum, aconite, ledebouriella root, Indian bread with hostwood, and Pueraria root) on serum nitric oxide (NO), 5-HT, and hemorheology in migraine patients.^[69]^ Eighty-eight patients with migraine were randomly divided into control and observation groups, with 44 patients in each group. The control group was administered lomerizine hydrochloride 5 mg orally twice daily for 4 weeks. The observation group was treated with the Xiongzhi decoction for 4 weeks. Changes in laboratory indices (ET-1, NO, and 5-HT) and hemorheology were detected. The headache symptom score was evaluated based on the degree of pain, duration, and attack frequency, and clinical efficacy was compared. Conclusion: Lomerizine hydrochloride combined with Xiongzhi decoction is effective in the treatment of migraine, which may be related to the upregulation of the expression of NO and 5-HT and the improvement of hemorheology.

## 10. Acupuncture for migraine

5-Factors such as HT, CGRP, and plasma substance P (SP) mediate different pathological processes at different stages of migraine. Acupuncture treatment for migraine also revolves around these humoral factors.

Zhao Zhen et al designed a RCT to intervene in vestibular migraine with Tongxuan acupuncture (Fengchi, Touwei, Baihui, Taixi, Taichong, Yinlingquan, and Fenglong). To observe the clinical efficacy and changes in the levels of the neurotransmitters 5-HT, CGRP, and SP.^[70]^ Eighty patients with vestibular migraine in our hospital were randomly divided into treatment and control groups at a 1:1 ratio. Both groups were administered with flunarizine hydrochloride capsules. The treatment group was treated with Tongxuan acupuncture, and the control group was treated with placebo acupuncture. The vertigo disability scale (dizziness handicap inventor), cumulative number and duration of vertigo attacks, and changes in neurotransmitter 5-HT, CGRP, and SP levels were compared between the 2 groups. Researchers have found that Tongxuan acupuncture has a significant effect on vestibular migraine and can effectively improve its clinical symptoms. Moreover, the Tongxuan needling method can shorten the treatment cycle and regulate the level of neurotransmitters in the brain, thus controlling the recurrence of vertigo.

Liu Shanshan et al Designed RCT.^[71]^ Based on resting-state functional magnetic resonance imaging, researchers have observed the effect of acupuncture on migraine without aura and changes in resting-state brain FC before and after acupuncture. Thirty-four patients with migraine without aura were selected as the observation group and treated with acupuncture in Baihui, Xuanlu, Shuaigu, and Taiyang. Fengchi and Shuaigu, on the same side, were connected to a G6805-II electroacupuncture instrument. Acupuncture was performed for 20 minutes each time, twice a week (interval > 2 days) for 6 weeks (12 times). Sixteen healthy sex- and age-matched healthy subjects without any acupuncture intervention were recruited as the control group. The number of headache days, visual analog scale (VAS) score of headache degree, total score of headache symptoms, migraine-specific quality of life questionnaire (MSQ) score, self-rating anxiety scale score, and self-rating depression scale score were compared before and after acupuncture treatment in the observation group, and clinical efficacy was evaluated. The resting state functional magnetic resonance data of the observation group before and after acupuncture treatment and the control group at baseline were collected, and the periaqueductal gray was used as the seed point to analyze the effect of acupuncture on the whole-brain FC of migraine patients without aura and the correlation between FC, VAS score, and headache days. The researchers found that acupuncture had a positive effect on migraine without aura, and there were abnormal changes in cerebral FC in patients with migraine without aura. Acupuncture may function by modulating dysfunctional brain regions and activating pain-related emotional brain regions.

Ke Hongkui et al designed an RCT to observe the clinical efficacy and mechanism of action of “Zhu Lian acupuncture and moxibustion inhibiting type II manipulation” in the treatment of chronic migraine.^[72]^ One hundred twenty patients with chronic migraine were randomly divided into the observation group (60 cases, 3 dropouts) and the control group (60 cases, 2 dropouts). The control group was administered 5 mg flunarizine hydrochloride capsules once daily. The patients in the observation group were treated with Zhu Lian acupuncture and moxibustion inhibiting type II manipulation, including Shousanli, Hegu, Yangbai, Tongziliao, and Zusanli once every other day. Both groups were treated for 4 weeks. The migraine clinical symptom scores, cerebral hemodynamics indices (blood flow velocity of the anterior cerebral artery, posterior cerebral artery, bilateral middle cerebral artery, and basilar artery), and serum related indices (5-HT, vascular endothelial growth factor [VEGF]), CGRP, and MSQ. Researchers have found that “Zhu Lian acupuncture and moxibustion inhibition of type II manipulation” can reduce the frequency and duration of migraine attacks in patients with chronic migraine, reduce the degree of pain, and improve the speed of cerebral blood flow and quality of life. This mechanism may be related to the regulation of serum 5-HT, CGRP, and VEGF levels.

Li Tao et al designed an RCT to observe the effect of “Dragon and Tiger Battle” acupuncture on confluence points of 8 vessels on headache days and CGRP expression level in patients with migraine without aura (MO).^[73]^ Methods: According to the degree of headache, 90 MO subjects were randomly divided into treatment group I, treatment group II, and control group, with 30 cases in each group. Treatment Group I was treated with needling the confluence points of the 8 vessels (Waiguan and Zulinqi) with the method of “battle between dragon and tiger.” Treatment group II was treated with acupuncture at the confluence points of the 8 vessels (Waiguan and Zulinqi), and the control group was treated with acupuncture at the non-meridian and non-acupoints, with the needle retained for 30 minutes each time, 5 times a week, for 20 times. Before treatment and 4 weeks after the end of treatment, the changes in headache days in the 3 groups were recorded to determine efficacy. The elbow veins of the patients were collected before treatment and 4 weeks after treatment. The CGRP content in the serum was detected by ELISA. Conclusion: The Results showed that (1) the method of “dragon and tiger fight” confluence points of the 8 vessels can significantly improve the number of headache days and decrease the expression level of serum CGRP in MO patients. This may be one of the mechanisms of acupuncture confluence points of the 8 vessels in treating MO. (2) The effect of acupuncture on non-meridian and non-acupoints was not persistent, while the effect of acupuncture on confluence points of the 8 vessels was persistent; (3) confluence points of the 8 vessels can give full play to the therapeutic effect of compound acupuncture manipulation. This suggests that we should not only pay attention to the compatibility of acupoints in the clinical treatment of diseases, but also that appropriate acupuncture manipulation can improve clinical efficacy.

## 11. Comprehensive treatment of migraine with traditional Chinese medicine

Ruihuan et al^[74]^ used meta-analysis to evaluate the clinical efficacy and safety of acupuncture combined with TCM in the treatment of migraine. Randomized controlled studies on acupuncture combined with TCM in the treatment of migraine were retrieved from the CNKI, Wanfang Academic Journal Full-text Database, VIP Chinese Science and Technology Journal Database, Chinese Biomedical Database, American Biomedical Information Retrieval System, and International Evidence-based Medicine Library. Patients in the control group were treated with flunarizine, whereas those in the experimental group were treated with acupuncture combined with TCM. The meta-analysis was performed using Review Manager version 5.3 software. The study found that the clinical cure rate and total effective rate of acupuncture combined with TCM in the treatment of migraine were higher than those of Sibelium, the VAS score, headache attack frequency, headache duration, and headache score were lower, and the incidence of adverse reactions was lower. Therefore, acupuncture and moxibustion combined with TCM are superior to sibelium in the treatment of migraine. At the same time, in the treatment of acupuncture combined with TCM, a blind operation cannot be achieved. Based on the lack of RCT trials and the characteristics of migraine, it is expected that future clinical research can standardize the evaluation scale of migraine, adopt the international unified scale of migraine evaluation, carry out large-sample, high-quality RCT, and long-term clinical research of acupuncture and moxibustion combined with TCM in the treatment of migraine, so as to improve the evidence level of clinical efficacy and prognosis.

Tang Yu et al designed an RCT to analyze the clinical efficacy of Danzhi Xiaoyao Powder and Ganmai Dazao Decoction combined with Shugan Tiaoshen acupuncture on migraine and its effects on TNF-α, intercellular adhesion molecule-1 (ICAM-1), and CGRP levels.^[75]^ In this study, 126 patients with migraine were randomly divided into a combination group (n = 63) and a control group (n = 63). Both groups were administered oral flunarizine hydrochloride tablets and acupuncture treatment for soothing the liver and regulating the mind, and the combined group was administered Danzhi Xiaoyao Powder and Ganmai Dazao Decoction for 1 month. Changes in headache symptoms and quality of life were compared between the 2 groups before and after treatment, and changes in cerebral hemodynamics and serum indexes were compared between the 2 groups before and after treatment. The study found that Danzhi Xiaoyao Powder and Ganmai Dazao Decoction applied in the treatment of migraine on the basis of soothing the liver and regulating the mind needling method not only helps to improve the headache symptoms, cerebral hemodynamics, and quality of life, but also improves the serum TNF-α, ICAM-1, and CGRP, so as to achieve a more ideal clinical effect.

Hongwei et al designed an RCT to observe the clinical efficacy of Wuzhuyu Tang combined with acupuncture at Shaoyang meridian points on migraine with cold coagulation and blood stasis syndrome.^[76]^ Ninety patients were randomly divided into control and observation groups. Eighty-one patients were included in the per protocol set. Wuzhuyu Tang (medicinal Evodia fruit, chuanxiong root, angelica root, and field mint) was orally administered to 41 patients in the control group. The observation group (40 cases) was treated with acupuncture at the points of Shaoyang meridian (Sizhukong, Shuaigu, Taiyang, Fengchi, Hegu, Taichong, Zulinqi, Yanglingquan and Waiguan) on the basis of the control group. Both groups could take the necessary analgesics at the same time. The frequency (times, days), duration, and degree of headache were observed at the beginning of treatment and at 4, 8, 12, and 4 weeks after treatment. Repeated-measures analysis of variance and generalized estimating equations were used for statistical analysis. Conclusion: Wuzhuyu Tang combined with acupuncture at the Shaoyang meridian points has a significant clinical effect on migraine with cold coagulation and blood stasis syndrome. The acupuncture scheme is simple, easy, easy to operate, and popular.

Wang Yue et al designed an RCT to observe the clinical efficacy of flunarizine capsule plus Xifeng Huatan Fang combined with electroacupuncture in the treatment of migraine of the wind-phlegm ascending type.^[77]^ A total of 110 cases of migraine of the wind-phlegm type were randomly divided into treatment and control groups, with 55 patients in each group. The patients in the control group were treated with flunarizine capsule combined with caltrop fruit, poria, white atractylodes rhizome, red tangerine peel, Pinellia rhizome, Angelica root, tall gastrodis tuber, chuanxiong root, gambir plant, Bupleurum, notoptetygium root, Corydalis rhizome, and licorice root). On the basis of the treatment in the control group, the treatment group was treated with electroacupuncture at the head and neck acupoints (bilateral Fengchi, Naokong, Shuaigu, and Tongziliao) of the Foot-Shaoyang Meridian. After 4 weeks of treatment, the clinical efficacy of the 2 groups was evaluated, and the changes in migraine symptom scores before and after treatment were observed, as well as the intracranial arterial blood flow velocity, including VA, basilar artery, anterior cerebral artery, middle cerebral artery, and posterior cerebral artery. Changes in serum nuclear factor-κB, cyclooxygenase-2, IL-1β, CGRP, ET-1, and NO levels were compared between the 2 groups before and after treatment. Conclusion: Flunarizine capsule plus Xifeng Huatan Decoction combined with electroacupuncture can effectively relieve migraine symptoms, reduce the average blood flow velocity of cerebral arteries, and downregulate the secretion of nuclear factor-κB, cyclooxygenase-2, and IL-1β. These effects may be related to improvements in vasomotor function.

Zheng et al designed an RCT to observe the clinical efficacy of Banxia Baizhu Tianma Tang combined with acupuncture in treating migraine gout with phlegm disturbance syndrome and its regulatory effect on neurovasoactive peptides and vascular endothelial activation mediators.^[78]^ A total of 150 patients were randomly divided into control and observation groups, with 75 cases in each group. Both groups were treated with acupuncture (Fengchi, Shuaigu, Taiyang, Xiaxi, Xingjian, Xiaxi, Taixi, and Sanyinjiao). The control group was administered Banxia Baizhu Tianma Tang placebo granules. The patients in the observation group were treated with Banxia Baizhu Tianma Tang (Pinellia rhizome, white Atractylodes rhizome, tall gastrodis tuber, Poria, red tangerine peel, chuanxiong root, Angelica root, Gambir plant, Corydalis rhizome, Bupleurum, notoptetygium root, Caltrop fruit, stiff silkorm, and licorice root). The course of treatment was 4 weeks in both groups. The VAS score was recorded after treatment, and pain relief, pain disappearance, and pain recurrence rates were calculated. The number of migraine attacks, duration of headache, and degree of headache were recorded 4 weeks before treatment, 4 weeks after treatment, and 4 weeks after treatment. The accompanying symptoms, wind-phlegm syndrome, HIT-6, and MIDAS scores were recorded before and after treatment, and the levels of CGRP, NO, ET-1, PACAP, S100B protein, SP, vWF, and FIB were measured before and after treatment. Simultaneously, safety evaluation was conducted. Conclusion: Banxia Baizhu Tianma Tang combined with acupuncture has a good immediate analgesic effect on migraine with wind-phlegm disturbance, has a significant effect on continuous analgesia, and reduces the recurrence of headache. It can also alleviate migraine and accompanying symptoms and reduce the impact of migraine on daily life and the degree of disability. The mechanism of action may be related to the regulation of neurovasoactive peptides and vascular endothelial substances.

Cong et al designed an RCT to observe the efficacy of Chuanxiong Chatiao San combined with acupuncture in treating acute migraine attacks (syndrome of wind-phlegm blocking collaterals) and its influence on neurovascular active mediators.^[79]^ A total of 134 patients who met the requirements were randomly divided into control and observation group with 67 cases. The basic treatment for the 2 groups was oral ibuprofen sustained-release tablets. The control group received acupuncture (Baihui, Shenting, Taiyang, Touwei, Jiaosun, Yintang, Fengchi, and Hegu). The acupuncture treatment in the observation group was the same as that of the control group and Chuanxiong Chatiao San (chuanxiong root, Schizonepeta, field mint, notoptetygium root, Asarum, angelica root, licorice root, Ledebouriella root, Pinellia rhizome, tall gastrodis tuber, Corydalis rhizome, and paniculate swallowwort root). VAS scores were evaluated before treatment, 1 day after treatment, and 2 to 10 days after treatment. Headache duration and degree, accompanying symptoms, frequency of headache attack, syndrome of wind-phlegm blocking collaterals, and migraine-specific quality of life scale scores were evaluated before and after treatment. Levels of NO, ET-1, CGRP, 5-HT, and β-endorphin were measured before and after treatment. Conclusion: Oral administration of Chuanxiong Chatiao San combined with acupuncture for the treatment of migraine patients with acute attacks has the same effect in terms of rapid pain relief. However, it has the advantages of an analgesic effect, lasting effect, low recurrence rate, high pain relief rate, and disappearance rate. It can also regulate neurovasculogenic active mediators, thus more effectively controlling acute attacks of migraine and improving the quality of life of patients.

Li Rui et al designed an RCT to observe the clinical efficacy of auricular venous bloodletting combined with auricular point sticking in the treatment of MM of qi stagnation and blood stasis type, and explored its possible mechanism of action.^[80]^ In this study, 102 patients were randomly divided into an observation group (51 cases, 3 dropouts) and a control group (51 cases, 2 dropouts). Bloodletting in the dorsal vein of the ear combined with auricular point sticking: bloodletting in the upper 1/3 of the dorsal vein of ear near the helix, and auricular point sticking alternately in the subcortex, endocrine, sympathetic, temporal, occipital, Shenmen, pancreatic-gallbladder, once every 7 days from 7 days before menstruation, 3 times as a course of treatment, for a total of 1 course of treatment, and auricular point sticking once every 3 days. The patients in the control group were treated with flunarizine hydrochloride capsules (2 capsules each time, once a day, from 7 days before menstruation, for 3 consecutive weeks). The headache index and VAS score were observed before treatment, after 1 menstrual cycle, and the first and second menstrual cycles after treatment in the 2 groups. The MSQ and the levels of serum estradiol (E2) and 5-HT were compared between the 2 groups before treatment and after 1 menstrual cycle, and the clinical efficacy was evaluated after 1 menstrual cycle. The study found that the combination of bloodletting and auricular point sticking could relieve the intensity of headache and improve the quality of life of patients with menstrual migraine of qi stagnation and blood stasis type, which may be achieved by upregulating the levels of serum E2 and 5-HT and improving the hormone levels in vivo.

## 12. Discussion

In summary, a combination of acupuncture and medicine is feasible for the treatment of migraine. This shows that TCM has great advantages in treating migraine and good development prospects. Many studies have shown^[59]^ that a variety of TCM treatments have significant effects on migraine, which can improve the degree of headache and the number of headache attacks. Some TCM treatments can improve the blood flow velocity of the cerebral arteries, thus alleviating headache symptoms. Thus, the therapeutic effect is definite. In addition, the application of TCM treatment has a high safety. The incidence of adverse reactions after medication is low, and it has a higher application advantage. These studies can improve the level of traditional Chinese used in the treatment of migraine.

However, there are still many problems in the study of TCM for the treatment of migraine. There are many studies on the treatment of migraine in TCM, but most of them are of low quality.^[81]^ Existing studies have shown that TCM has certain efficacy and advantages in the treatment of migraine, but there are still some shortcomings: ① TCM is mostly preventive treatment of migraine, but there are few studies on the acute attack of migraine; ② most of the studies are not rigorous enough, most of the randomized methods are not clear, most of the blind methods are not mentioned or non-blind trials; there are some differences in the inclusion and exclusion criteria of ③, and the reference diagnostic criteria are not completely consistent, especially the diagnostic criteria and syndrome differentiation of TCM, which lack of a unified standard; the TCM syndrome types of ⑤ migraine mainly include the syndrome of cold coagulation in liver vessels, the syndrome of hyperactivity of liver-yang, the syndrome of wind-phlegm disturbance, the syndrome of blood stasis in collaterals, the syndrome of deficiency of qi and blood, and the syndrome of deficiency of liver and kidney, while the drug treatment mainly focuses on the syndrome of hyperactivity of liver-yang, the syndrome of wind-phlegm disturbance, and the syndrome of blood stasis in collaterals, and less attention is paid to the syndrome of deficiency of liver and kidney and the syndrome of cold coagulation in liver vessels. ⑥ Curative effect index: The severity and attack frequency of migraine are often used as the curative effect index in the world, while existing studies in China only use 1 clinical total effective rate as the curative effect index. Some study outcome measures were so different that they could not be reviewed systematically. Randomized, double-blind, placebo-controlled, multicenter, large-sample clinical studies are required in the future. Strict inclusion criteria were set according to the unified diagnostic criteria, and international efficacy criteria were adopted. Some studies have compared the difference in curative effects between treatments based on syndrome differentiation and treatment based on disease differentiation. In the future, we can further explore the difference in curative effect between treatment based on syndrome differentiation and treatment based on disease differentiation. At present, this is mainly preventive research, and more acute-phase research can be carried out in the future. In particular, the combination of acupuncture and medicine has a good effect on acute headaches, and more related studies can be carried out.

The purpose of this review is to collect data for the team to conduct high-quality RCT in strict accordance with the blind method in the future. In the next step, the team will conduct a meta-analysis to systematically evaluate the clinical efficacy and safety of acupuncture combined with medicine for the treatment of migraine. Finally, based on these studies, the team will design a randomized controlled trial to explore the combination of acupuncture and medicine in the treatment of migraine.

## Author contributions

**Conceptualization:** Yue Shen.

**Data curation:** Yue Shen, Zeguang Li, Jing Wang, Zitong Qiu.

**Formal analysis:** Yue Shen.

**Funding acquisition:** Yue Shen.

**Investigation:** Yue Shen.

**Methodology:** Yue Shen.

**Project administration:** Yue Shen.

**Resources:** Yue Shen.

**Software:** Yue Shen.

**Supervision:** Yue Shen.

**Visualization:** Yue Shen.

**Writing – original draft:** Yue Shen.

**Writing – review & editing:** Yue Shen.
